# Lethal ventricular arrhythmia accompanied with myopalladin truncation mutation: a case report

**DOI:** 10.1093/ehjcr/ytag228

**Published:** 2026-03-20

**Authors:** Daiki Yamashita, Ryuji Okamoto, Yoshihiko Kagawa, Yoshihiro Asano, Kaoru Dohi

**Affiliations:** Department of Cardiology and Nephrology, Mie University Graduate School of Medicine, 2-174 Edobashi, Tsu, Mie 514-8507, Japan; Department of Cardiology and Nephrology, Mie University Graduate School of Medicine, 2-174 Edobashi, Tsu, Mie 514-8507, Japan; Regional Medical Support Center, Mie University Hospital, 2-174 Edobashi, Tsu, Mie 514-8507, Japan; Department of Clinical Training and Career Support Center, Mie University Hospital, 2-174 Edobashi, Tsu, Mie 514-8507, Japan; Department of Cardiology and Nephrology, Mie University Graduate School of Medicine, 2-174 Edobashi, Tsu, Mie 514-8507, Japan; Department of Cardiovascular Medicine, Osaka University Graduate School of Medicine, 2-2 Yamadaoka, Suita, Osaka 565-0871, Japan; Department of Genome Medicine, National Cerebral and Cardiovascular Center, 6‐1 Kishibe‐Shimmachi, Suita, Osaka 564‐8565, Japan; Department of Cardiology and Nephrology, Mie University Graduate School of Medicine, 2-174 Edobashi, Tsu, Mie 514-8507, Japan

**Keywords:** arrhythmogenic left ventricular cardiomyopathy, Case report, Myopalladin, Truncating mutation, Ventricular fibrillation, Subcutaneous ICD

## Abstract

**Background:**

Myopalladin (MYPN) is a structural protein in the Z-disk that plays an important role in mechanotransduction and signalling to the nucleus. Although MYPN mutations have been reported in various cardiomyopathies, their association with arrhythmogenic phenotypes and malignant ventricular arrhythmias remains poorly characterized.

**Case summary:**

A 25-year-old man developed ventricular fibrillation (VF) while at a golf driving range. The patient had a family history of sudden death in a maternal uncle and grandfather. Examinations were performed to determine the cause of VF, such as acute coronary syndrome, vasospastic angina, or concealed long QT syndrome, but no obvious cause was found. However, mild, diffuse impairment of left ventricular (LV) wall motion without LV dilatation was observed on transthoracic echocardiography and magnetic resonance imaging. A subcutaneous implantable cardioverter defibrillator was implanted. Whole-exome linkage analysis revealed an MYPN R763X truncating mutation in both the patient and his mother. The patient was diagnosed with arrhythmogenic left ventricular cardiomyopathy (ALVC) in the presence of a maternally inherited MYPN truncating mutation. No significant abnormalities were apparent in other genes associated with VF or cardiomyopathies.

**Discussion:**

This case suggests a possible involvement of MYPN truncating mutations in ALVC.

Learning points

- Arrhythmogenic left ventricular cardiomyopathy can be accompanied only by a mildly reduced left ventricular ejection fraction.
- Myopalladin is one of the candidate genes associated with dilated cardiomyopathy.

## Introduction

Arrhythmogenic cardiomyopathy (ACM) is a hereditary disease of the cardiac muscle characterized by fibrofatty tissue replacing ventricular myocardium.^1^ The current classification of ACM includes major phenotypes of arrhythmogenic right ventricular (RV) cardiomyopathy with predominant RV involvement and no left ventricular (LV) abnormalities, arrhythmogenic ventricular cardiomyopathy with equal involvement of both ventricles, and arrhythmogenic LV cardiomyopathy (ALVC) with predominant LV involvement and no or only minor RV abnormalities.^2^ Recent genetic analyses have indicated that mutations such as those in the lamin and RBM20 genes are common in ALVC, but other genes are not well known.^3^  *MYPN* codes myopalladin (MYPN), an important structural member of the Z-disk, and mutations have recently been reported in patients with dilated cardiomyopathy (DCM).^4^ Although MYPN mutations have been reported in various cardiomyopathies, their association with arrhythmogenic phenotypes and malignant ventricular arrhythmias remains poorly characterized. We describe herein a case of ALVC with diffusely impaired LV systolic function and complicated by lethal ventricular arrhythmia.

## Summary figure

**Figure:**
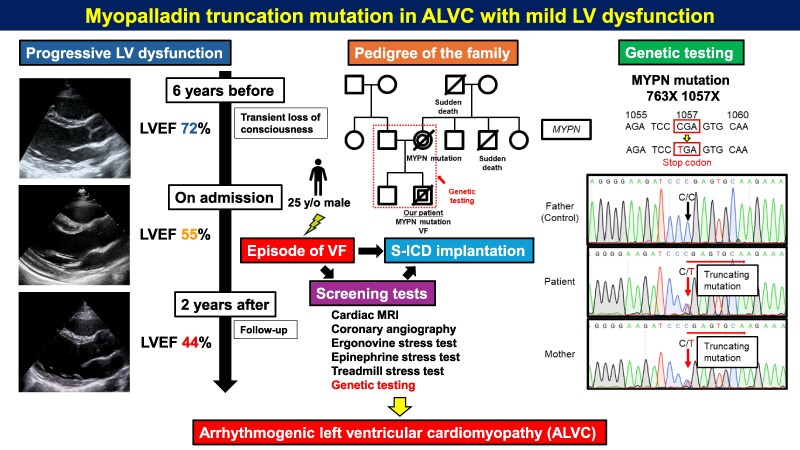


## Case report

**Figure 1:**
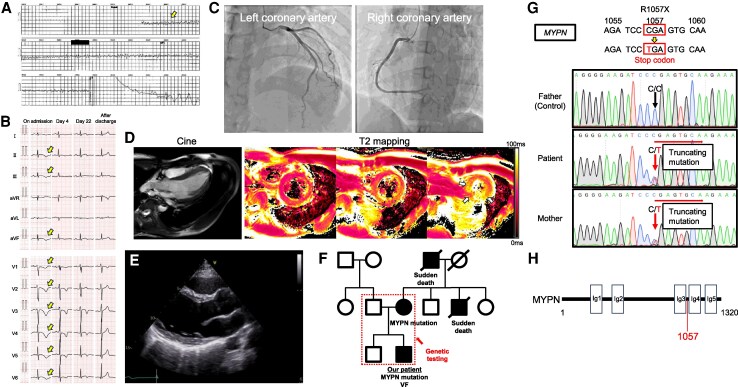
Multimodal imaging demonstrating the findings in this case. (*A*) Ventricular fibrillation documented by the automated external defibrillator (yellow arrows). (*B*) Electrocardiogram (ECG) on admission shows sinus rhythm, negative T waves in leads II, III, aVF, and V1–6, and QT prolongation (QTc; 569 ms) with a heart rate of 76 beats/min (yellow arrows). Follow-up ECG shows no abnormal findings, compared to on admission. (*C*) Emergency coronary angiography performed on the same day shows no significant coronary artery stenosis. (*D*) Cardiac magnetic resonance imaging with contrast shows evidence of left ventricular dysfunction and myocardial oedema at T2 (global T2: 64 ms; normal range: 46 ± 4 ms, white arrows). Left ventricular (LV) ejection fraction is decreased (38.0%) without LV dilatation. (*E*) Follow-up transthoracic echocardiography shows normal global contraction, but mildly and diffusely decreased LV wall motion remains. (*F*) Family tree of the patient. Squares denote male family members, circles denote female members, and diagonal lines indicate members with various diseases. Double lines indicate members with apparent myopalladin (MYPN) mutation. (*G*) Partial sequence electropherogram of MYPN with nucleotide substitution in the two affected members analysed. An MYPN R1057X (CGA → TGA; stop codon) truncating mutation is documented. (*H*) MYPN mutation identified in the present case. The upper panel shows a schematic presentation of MYPN structure. Boxes indicate immunoglobulin (Ig)-like domain. The lower panel shows the position of the identified mutation (red). ECG = electrocardiogram; LV = left ventricular; MYPN = myopalladin.

A 25-year-old man was transported to the emergency room of another hospital after experiencing cardiac arrest at a golf driving range. Bystander cardiopulmonary resuscitation had been performed. Defibrillation was performed after ventricular fibrillation (VF) was documented by an automated external defibrillator (*Figure 1A*). Return of spontaneous circulation (ROSC) was achieved; then, the patient was intubated and transferred to our hospital for intensive care. The patient had a family history of sudden deaths in a maternal uncle while walking to work at 24 years old and a maternal grandfather while taking a bath at 83 years old. The patient had a medical history of transient loss of consciousness at 20 years old, which had been investigated closely on suspicion of epilepsy. Full examinations failed to find any obvious abnormalities, and the patient was followed up. Physical examination revealed: heart rate, 123 beats/min; blood pressure, 92/66 mmHg; and oxygen saturation, 100% (fraction of inspired oxygen = 0.4). Glasgow Coma Scale score was E1VTM4. Chest X-ray showed mild congestion, with a cardiothoracic ratio of 55%. Electrocardiogram (ECG) showed sinus rhythm, negative T wave in II, III, aVF, and V1–6 leads, and QT prolongation (QTc, 569 ms) with a heart rate of 76 beats/min (*Figure 1B*). Laboratory tests revealed elevated troponin I (1551.8 pg/mL; normal, <34.2 pg/mL), and concentrations of B-type natriuretic peptide level (9.5 pg/mL) and potassium (4.8 mmol/L) within normal ranges. Transthoracic echocardiography (TTE) showed diffuse mild LV hypokinesis, particularly as decreased regional wall motion at the LV apex [LV ejection fraction (LVEF), 40%], an interventricular septal thickness of 11 mm, and a posterior wall thickness of 11 mm. Emergency coronary angiography was performed on the same day and showed no significant coronary artery stenosis (*Figure 1C*). A loading dose of amiodarone had been initiated at the referring hospital but was discontinued after transfer to our institution. Targeted temperature management was then performed. The patient was subsequently extubated, and no neurological sequelae were identified. Thereafter, no fatal arrhythmia appeared, and QT prolongation on ECG gradually improved (*Figure 1B*).

On hospital day 10, contrast-enhanced cardiac magnetic resonance imaging (CMR) showed that LVEF was decreased (38.0%) without LV dilatation, with a left ventricular end-diastolic volume of 178 mL, an end-systolic volume of 110 mL, and a left ventricular mass of 101 g (*Figure 1D*, Supplementary material online, *Video S1*). CMR was performed in accordance with current guidelines to evaluate underlying cardiomyopathy, myocardial inflammation, and arrhythmogenic substrate in survivors of unexplained VF. No late gadolinium enhancement (LGE) was observed. Native T1 was mildly prolonged (1360 ms; normal, 1314 ± 29 ms); extracellular volume was 31% (normal, 26.8 ± 5%); and evidence of myocardial aedema was seen at T2 (global T2, 64 ms; normal, 46 ± 4 ms) (*Figure 1D*). On hospital day 14, an ergonovine stress test was also performed to evaluate the possibility of vasospastic angina, in line with guideline-based diagnostic approaches in patients with VF without obstructive coronary artery disease, yielding a negative result. An epinephrine stress test was also performed to evaluate the possibility of concealed long QT syndrome (LQTS), as recommended by current guidelines for the evaluation of survivors of unexplained cardiac arrest, again with a negative result. On hospital day 18, a subcutaneous implantable cardioverter defibrillator (S-ICD) was implanted for secondary prevention of sudden cardiac death, in accordance with current guideline recommendations for survivors of VF without a reversible cause. Genetic testing was performed, and the patient was discharged home on hospital day 27.

During three and a half years of follow-up, no lethal ventricular arrhythmias have been observed, and no appropriate or inappropriate therapies were recorded by the S-ICD. The patient remained clinically stable and asymptomatic (New York Heart Association functional class I). Follow-up ECG showed no abnormal findings such as QT prolongation, compared to admission (*Figure 1B*). No arrhythmia or waveform changes were observed on ECG during the treadmill stress test. We recognized mild and diffuse impairment of LV wall motion without LV dilatation on follow-up TTE (LVEF, 44%; *Figure 1E*, Supplementary material online, *Video S2*) compared with six years before admission (LVEF, 72%; Supplementary material online, *Video S3*). Genomic linkage analysis by whole exome sequencing revealed an MYPN R1057X (CGA → TGA; stop codon) truncating mutation in both the patient and his mother (*Figures 1F–1H*). This variant is classified as pathogenic by ACMG (PVS1PM2PP3, Minor Allele Frequency = 0.0000021). No significant abnormalities were apparent in other genes associated with lethal arrhythmia or cardiomyopathies. The patient was diagnosed with ALVC in the presence of a maternally inherited truncating MYPN variant. The patient has since been followed at an outpatient clinic.

## Discussion

MYPN is a structural protein in the Z-disk that plays an important role in mechanotransduction and signalling to the nucleus. Some reports have described that MYPN gene mutations are associated with cardiomyopathy, including DCM, hypertrophic cardiomyopathy, and restrictive cardiomyopathy.^5,6^ A previous report also showed that double missense mutations in MYPN and myosin binding protein C could be associated with DCM complicated with diffuse coronary artery disease and complete atrioventricular block in humans.^5^ In addition to cardiomyopathy, it has been reported that the MYPN homozygous truncation mutation is associated with slowly progressive skeletal myopathy, which of onset was in the first decade,^6^ although the present case was heterozygous and did not show any muscle weakness.

The patient developed VF while at a golf driving range. Examinations were performed to identify the cause of VF, such as acute coronary syndrome, vasospastic angina, LQTS, or other cardiomyopathies. The diagnostic workup and therapeutic strategy in this case, including CMR, pharmacological stress testing, and S-ICD implantation, were consistent with contemporary guideline recommendations for the evaluation and management of survivors of unexplained VF.^7^ In this case, no obvious cause was found. Initially, QT prolongation and diffuse LV hypokinesis were observed, but improved spontaneously over time. The acute phase after ROSC was considered to affect these findings, and the initial QT prolongation was likely related to transient metabolic derangements after return of spontaneous circulation. Myocarditis was considered but was deemed unlikely due to the absence of prodromal infectious symptoms, normal inflammatory markers, lack of myocardial oedema or inflammatory LGE on CMR, and spontaneous recovery of LV function. Stress cardiomyopathy was also considered; however, the absence of a typical trigger, nonclassic wall motion abnormalities, and VF as the initial presentation, together with the presence of a pathogenic truncating MYPN mutation, argued against this diagnosis.

The patient was therefore initially diagnosed with idiopathic VF. However, he had a family history of sudden deaths in a maternal uncle at 24 years old and a maternal grandfather at 83 years old. Moreover, genomic linkage analysis showed a MYPN truncating mutation in the patient and his mother. Mild, diffuse impairment of LV wall motion without LV dilatation was also noted on follow-up TTE. The patient was therefore thought to have ALVC, based on previous reports.^2,3^ However, given the wide age difference, a shared aetiology for these events cannot be definitively established. Instead, the family history raises suspicion of an inherited predisposition to malignant ventricular arrhythmias with variable age of onset and incomplete penetrance. In inherited cardiomyopathies and arrhythmia syndromes, including ACM, age-dependent penetrance and variable expressivity are well recognized, and sudden cardiac death may occur across a broad age range. Accordingly, the family history was considered supportive but not diagnostic of a shared aetiology.

Genetic analysis using a targeted cardiomyopathy gene panel identified a heterozygous truncating MYPN variant (R1057X) in the patient and his mother. This loss-of-function variant is extremely rare in population databases and was classified as pathogenic by InterVar according to ACMG criteria. No other pathogenic or uncertain variants associated with cardiomyopathy or inherited arrhythmia syndromes were detected. Although MYPN is not an established causative gene for ALVC, truncating MYPN variants have been reported in various cardiomyopathy phenotypes and may confer mechanical and electrical vulnerability of the myocardium. In this case, the combination of a pathogenic MYPN variant, recurrent VF, and a family history of sudden death supports a plausible genotype–phenotype association, although causality cannot be confirmed from a single case.

The diagnosis of ALVC was based on a comprehensive evaluation according to contemporary frameworks, including the Padua criteria,^2^ incorporating malignant ventricular arrhythmias, mild LV systolic dysfunction without dilatation, abnormal myocardial tissue characterization on CMR, and family history. Despite the absence of LGE, the overall findings were considered compatible with an ALVC phenotype. Nevertheless, this association should be interpreted cautiously, and further genotype–phenotype data are required.

No previous reports have associated a MYPN truncating mutation with lethal ventricular arrhythmia. As seen in this case, MYPN gene mutations are possibly associated with regional wall motion abnormalities and lethal ventricular arrhythmias such as VF. MYPN abnormalities may be taken into consideration, particularly when we see patients with mild LV dysfunction complicated by lethal arrhythmia. Moreover, eliciting the family history of cardiovascular disease or sudden death is crucial, along with any history of suspected cardiogenic syncope.

This case report has several limitations. First, transient myocardial stunning after VF and resuscitation may have contributed to the early LV systolic dysfunction observed in the acute phase. Second, histological confirmation of fibrofatty myocardial replacement was not available, as no endomyocardial biopsy was performed. Third, although a truncating MYPN mutation was identified, genetic evidence supporting a direct association between MYPN truncating variants and malignant ventricular arrhythmias remains limited, and a causal relationship cannot be established from a single case. If similar observations are confirmed in future reports, a potential causal relationship between MYPN truncating variants and lethal ventricular arrhythmia could be hypothesized. Fourth, phenotypic evaluation of family members was incomplete; the patient’s mother, despite carrying the same variant, was asymptomatic and did not undergo detailed cardiac imaging. Finally, MYPN variants have only rarely been reported in association with arrhythmogenic phenotypes, highlighting an important knowledge gap that this case seeks to address.

## Conclusion

This case suggests a possible involvement of MYPN truncating mutations in ALVC. Abnormalities of MYPN may be taken into consideration, particularly when we see patients with mild LV dysfunction complicated by lethal arrhythmia. Moreover, it is important to ask about the family history of cardiovascular disease or sudden death, and the history of syncope suspected to be cardiogenic.

## Lead author biography


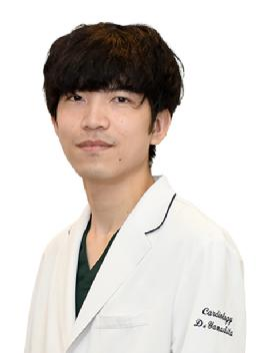


Daiki Yamashita is a cardiologist at Mie University Hospital, Mie, Japan. He is currently undertaking a PhD with Mie University, exploring arrythmia and cardiomyopathy studies in Mie prefecture.

## Supplementary Material

ytag228_Supplementary_Data

## Data Availability

The patient’s data will not be shared.

## References

[1] Corrado D, Basso C, Judge DP. Arrhythmogenic cardiomyopathy. Circ Res 2017;121:784–802. 28912183 10.1161/CIRCRESAHA.117.309345

[2] Corrado D, Basso C. Arrhythmogenic left ventricular cardiomyopathy. Heart 2022;108:733–743. 34257076 10.1136/heartjnl-2020-316944 PMC8995901

[3] Lukas Laws J, Lancaster MC, Ben Shoemaker M, Stevenson WG, Hung RR, Wells Q, et al Arrhythmias as presentation of genetic cardiomyopathy. Circ Res 2022;130:1698–1722. 35617362 10.1161/CIRCRESAHA.122.319835 PMC9205615

[4] Duboscq-Bidot L, Xu P, Charron P, Neyroud N, Dilanian G, Millaire A, et al Mutations in the Z-band protein myopalladin gene and idiopathic dilated cardiomyopathy. Cardiovasc Res 2008;77:118–125. 18006477 10.1093/cvr/cvm015

[5] Mastroianno S, Palumbo P, Castellana S, Leone MP, Massaro R, Potenza DR, et al Double missense mutations in cardiac myosin-binding protein C and myopalladin genes: a case report with diffuse coronary disease, complete atrioventricular block, and progression to dilated cardiomyopathy. Ann Noninvasive Electrocardiol 2020;25:e12687. 31524317 10.1111/anec.12687 PMC7358828

[6] Miyatake S, Mitsuhashi S, Hayashi YK, Purevjav E, Nishikawa A, Koshimizu E, et al Biallelic mutations in MYPN, encoding myopalladin, are associated with childhood-onset, slowly progressive nemaline myopathy. Am J Hum Genet 2017;100:169–178. 28017374 10.1016/j.ajhg.2016.11.017 PMC5223057

[7] Zeppenfeld K, Tfelt-Hansen J, de Riva M, Winkel BG, Behr ER, Blom NA, et al 2022 ESC guidelines for the management of patients with ventricular arrhythmias and the prevention of sudden cardiac death. Eur Heart J 2022;43:3997–4126. 36017572 10.1093/eurheartj/ehac262

