# Supplementary material for: Lethal ventricular arrhythmia accompanied with myopalladin truncation mutation: a case report
Source: Eur Heart J Case Rep. 2026 Mar 20;10(4):ytag228. doi: 10.1093/ehjcr/ytag228 (PMC13062368; doi:10.1093/ehjcr/ytag228)
Supplement: ytag228_Supplementary_Data [file ytag228_supplementary_data.zip › Supplemental table (timeline) revise 1st 20260123.docx]

**Supplemental table**

**Timeline of the Case**

| **Timeline** | **Events** |
| --- | --- |
| Date of Submission/Day 1  (Another hospital) | 25 year-old man experienced sudden collapse due to VF.  Return of spontaneous circulation was achieved.  The patient was intubated and amiodarone administration was initiated. |
| Day 1  (Our hospital) | Transferred to our hospital. Initial ECG showed transient QT prolongation post-resuscitation. Echocardiography revealed mild LV systolic dysfunction without dilation. Emergency coronary angiography showed no significant stenosis. |
| Hospital course | Amiodarone discontinued. Targeted temperature management initiated and extubated. QT prolongation resolved spontaneously. |
| Day 10 | CMR revealed elevated native T1 and T2 values, without myocardial edema or LGE suggestive of myocarditis or ischemic injury. |
| Day 14 | Ergonovine stress test for vasospastic angina was negative.  Epinephrine stress test was also negative. |
| Day 18 | S-ICD was implanted. |
| Hospital course | Genetic testing was performed. |
| Day 27 | The patient was discharged home. |
| Genetic analysis | Targeted next-generation sequencing of genes associated with inherited cardiomyopathies and arrhythmia syndromes identified a heterozygous truncating MYPN variant (R1057X). The same variant was detected in the patient’s mother, consistent with autosomal dominant inheritance. No other pathogenic, likely pathogenic, or variants of uncertain significance were identified. |
| Variant interpretation | The MYPN truncating variant was classified as pathogenic according to ACMG criteria using InterVar, consistent with a loss-of-function mechanism. |
| Diagnosis | Based on recurrent VF, mild global LV systolic dysfunction without dilatation, abnormal myocardial tissue characterization (elevated T1/T2), family history of sudden cardiac death, and exclusion of alternative diagnoses, ALVC was diagnosed in accordance with contemporary diagnostic frameworks, including the Padua criteria. |
| Follow-up | During follow-up, no recurrent ventricular arrhythmias or ICD therapies were recorded. Mild LV systolic dysfunction persisted on echocardiography. QT intervals remained within the normal range. |

VF = ventricular fibrillation; ECG = Electrocardiogram; LV = left ventricular; CMR = cardiac magnetic resonance imaging; LGE = late gadolinium enhancement; S-ICD = subcutaneous implantable cardioverter defibrillator; MYPN = myopalladin; ACMG = American College of Medical Genetics and Genomics; ALVC = arrhythmogenic left ventricular cardiomyopathy.
